# Discovery: an interactive resource for the rational selection and comparison of putative drug target proteins in malaria

**DOI:** 10.1186/1475-2875-8-178

**Published:** 2009-07-30

**Authors:** Fourie Joubert, Claudia M Harrison, Riaan J Koegelenberg, Christiaan J Odendaal, Tjaart AP de Beer

**Affiliations:** 1 Bioinformatics and Computational Biology Unit, Department of Biochemistry, University of Pretoria, Pretoria 0001, South Africa

## Abstract

**Background:**

Up to half a billion human clinical cases of malaria are reported each year, resulting in about 2.7 million deaths, most of which occur in sub-Saharan Africa. Due to the over-and misuse of anti-malarials, widespread resistance to all the known drugs is increasing at an alarming rate. Rational methods to select new drug target proteins and lead compounds are urgently needed. The Discovery system provides data mining functionality on extensive annotations of five malaria species together with the human and mosquito hosts, enabling the selection of new targets based on multiple protein and ligand properties.

**Methods:**

A web-based system was developed where researchers are able to mine information on malaria proteins and predicted ligands, as well as perform comparisons to the human and mosquito host characteristics. Protein features used include: domains, motifs, EC numbers, GO terms, orthologs, protein-protein interactions, protein-ligand interactions and host-pathogen interactions among others. Searching by chemical structure is also available.

**Results:**

An *in silico* system for the selection of putative drug targets and lead compounds is presented, together with an example study on the bifunctional DHFR-TS from *Plasmodium falciparum*.

**Conclusion:**

The Discovery system allows for the identification of putative drug targets and lead compounds in Plasmodium species based on the filtering of protein and chemical properties.

## Background

Even after a century of attempts to control and eradicate malaria, up to half a billion clinical cases are reported each year [1] with an annual death rate of 2.7 million [2]. One of the most studied pathogens of all time, the parasite *Plasmodium falciparum *accounts for 80% of clinical cases and 90% of deaths from malaria [3]. Malaria is a complicated, but curable and preventable disease. Millions of lives can be saved if the disease is detected early and adequately treated. As *P. falciparum *is becoming increasingly resistant to current anti-malarial drugs and the development of a vaccine is an extremely time-consuming, ongoing process, the death rate is expected to double in the next 20 years [4]. Since malaria drug research is not economically feasible for international pharmaceutical companies, the responsibility to ease the burden of malaria falls upon the shoulders of third world countries.

A major limitation in malaria research is the availability of effective approaches to mine post-genomic data for new putative drug targets and lead compounds. The selection criteria for promising drug targets are therefore of extreme importance to researchers involved in malaria target screening projects and lead design [5,6]. Currently, the main available sources of protein annotation and interaction data specifically relevant to malaria include PlasmoDB [7,8], PlasmoMap [9,10], the Malaria Drug Targets database [11], the TDR Targets Database [12,13] and the *Plasmodium falciparum *Interactome Database [14].

Although PlasmoDB is a comprehensive annotation database, the focus of the search environment is not specifically directed towards the drug target and lead discovery process, and protein-ligand interactions are not provided. The MDT database does provide some information on potential drug targets and their inhibitors. PlasmoMap allows protein-protein interaction data from predictions to be downloaded, a single PlasmoDB ID may also be queried for predicted interactions, but no advanced searches against the data are offered. The TDR Targets database serves as an information repository as well as a tool for the prioritization of targets in whole genomes against neglected tropical diseases including malaria. PlasmoID is a developing project that provides protein interactions for *P. falciparum*, but the main aim of the project is to build metabolic pathways using protein-protein interactions. A brief summary of key features of these resources is provided in Table 1.

**Table 1:** The availability of some key features in malaria web resources relevant to drug target and lead identification.

	**Discovery**	**PlasmoDB**	**PlasmoMap**	**MDT**	**TDR**	**PlasmoID**
**Orthology**	✓	✓	✗	✗	✓	✗
**Functional Annotation**	✓	✓	✗	✓	✓	✓
**Metabolism**	✓	✓	✗	✗	✓	✗
**Structure**	✓	✓	✗	✓	✓	✗
**Protein Interactions**	✓	✓	✓	✗	✗	✓
**Druggability**	✗	✗	✗	✗	✓	✗
**Ligand Interactions**	✓	✗	✗	✗	✓	✗
**Host/pathogen**	✓	✗	✗	✗	✗	✗
**Expression**	✗*	✓	✗	✗	✓	✗
**Chemo-informatics**	✓	✗	✗	✗	✗	✗
**Literature**	✗*	✓	✗	✓	✓	✗

* In development

No tool exists to perform advanced interactive mining of protein together with ligand properties in a species-comparative environment. The Discovery project attempts to provide an interface where researchers may efficiently perform the selection of putative drug targets and lead compounds based on relevant properties. Relationships were constructed between the proteins, protein features and chemical compounds in a way that allows powerful searching and efficient navigation between molecules.

## Methods

**Figure 1:**
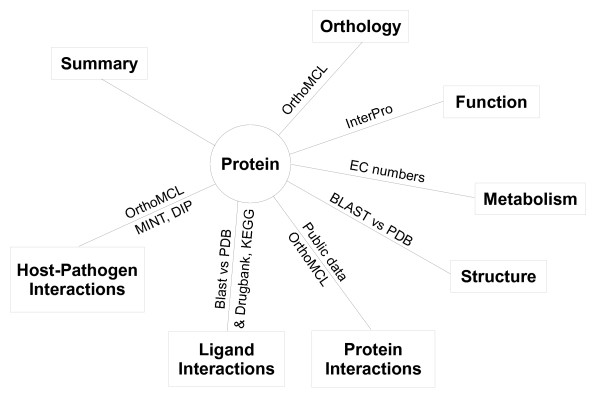
**A represenation of the major protein features displayed in the Discovery system, together with the sources of information**.

Discovery was developed in Python using the TurboGears web development framework [15] with an underlying MySQL database [16]. Protein sequences for *P. falciparum*, *Plasmodium vivax*, *Plasmodium chabaudi*, *Plasmodium berghei *and *Plasmodium yoelii *were obtained from PlasmoDB and for *Homo sapiens *and *Anopheles gambiae *from Ensembl [17]. Data for ligand-protein interactions were collected from DrugBank [18], KEGG [19] and PDB-Ligand [20] and protein-protein interaction data from MINT [21] and DIP [22]. Currently, only hits common to both MINT and DIP are displayed, with a MINT cut-off value of 0.3. Blast searches against PDB-Ligand and DrugBank were performed using NCBI BLAST with an E-value cut-off of 1.0e^-6^. InterPro [23] analysis was performed using the latest InterProScan [24] from the EBI. Homology models of *P. falciparum *and human proteins were obtained from ModBase [25]. Custom ortholog clusters were generated with OrthoMCL [26]. An outline of the structure of the Discovery system is provided in Figure 1. Chemical searches, displays and ADMET calculations were implemented using the Marvin applet and JChem Base from ChemAxon [27]. Jmol [28] was used to display the 3D structures and Jalview [29] to display the T-Coffee [30] alignments of the OrthoMCL clusters.

The power of the Discovery system is in the rich relations between the different data types in the system. Relations to protein features were created based on existing annotations, or analyses performed in-house. Inter-and intra-species protein-protein interaction predictions were based on orthology to interacting partners in DIP and MINT. Predicted protein-ligand interactions were based on KEGG annotations and on BLAST searches against PDB and DrugBank.

## Results and discussion

### A brief introduction to the resource

The Discovery resource was designed to provide an effective interface where researchers could view annotations for malaria proteins that are relevant to the drug design process, in an environment where comparisons could efficiently be made between organisms. Users may embark on a search from either a protein or ligand perspective.

**Figure 2:**
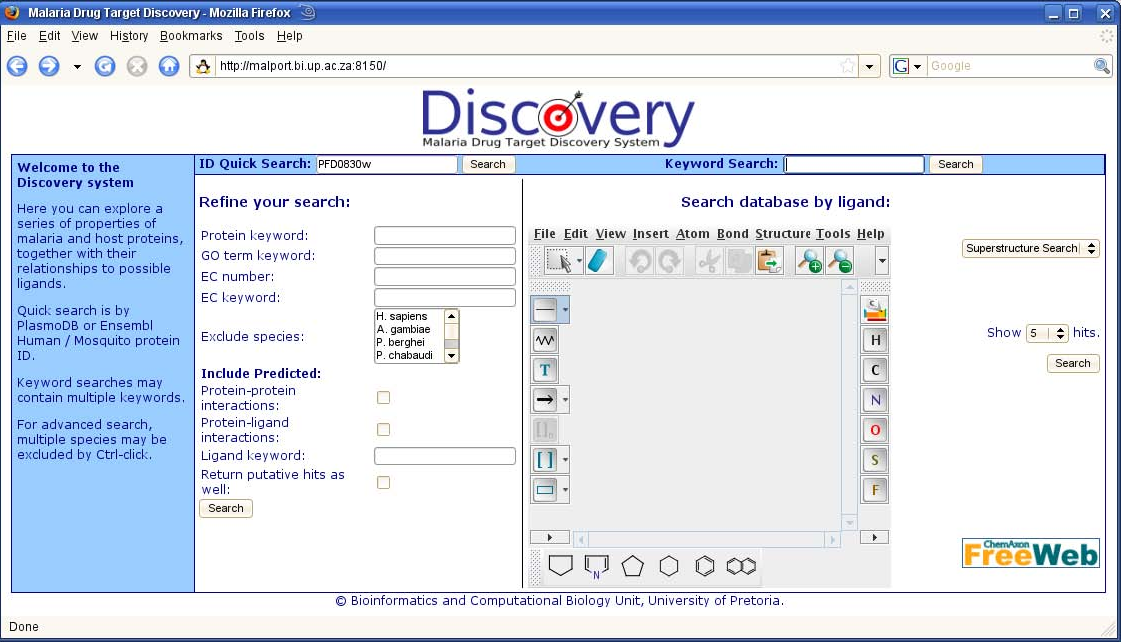
**The main Discovery interface, allowing ID, keyword, advanced protein property and chemical structure searches**.

**Figure 3:**
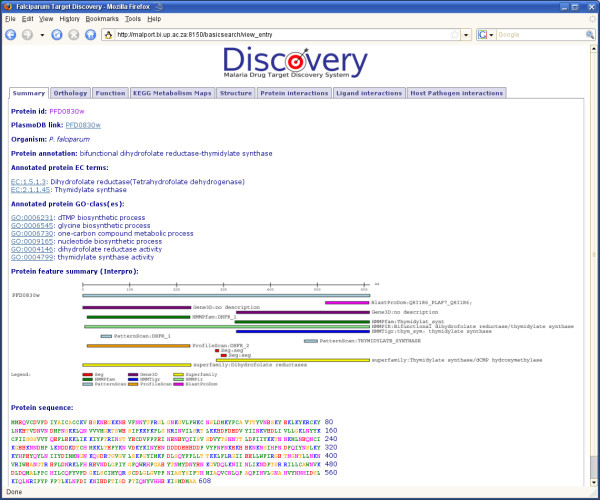
**The Discovery summary page, showing the tabbed environment for the different result classes**.

When performing a simple search from a protein perspective, users may enter a PlasmoDB/Ensembl ID or a keyword. The user is then presented with either the specified protein, or a list of proteins matching the keyword by description or by one of its relationships. This list is separated by species. The user is then able to further explore the hits of interest. More powerful is the advanced search function, where the user may enter search terms in several categories, and select for the presence or absence of specific relations (Figure 2). The results are returned as above. When exploring the data for a selected protein, the user is presented with a tabbed environment with different categories of data (Figure 3). The first tab shows a summary of the protein's GO and EC terms, a graphical summary of the InterPro features and a view of the protein sequence. Next is a view of the orthologs and paralogs predicted by OrthoMCL, together with a Jalview display of the T-Coffee alignment of these sequences. This is followed by the InterPro analysis results of the protein sequence, the position of the protein in the KEGG metabolic pathway maps and tertiary structure information such as ModBase models or BLAST results against PDB. The protein interaction tab provides results of possible protein-protein interactions based on Y2H studies, PlasmoMAP interactions and orthology to DIP and MINT interacting partners. The ligand interaction tab provides predicted ligand interactions based on KEGG annotations and BLAST results against PDB and DrugBank. Host-pathogen interaction predictions are based on orthology to MINT and DIP interacting partners, with species filtering.

**Figure 4:**
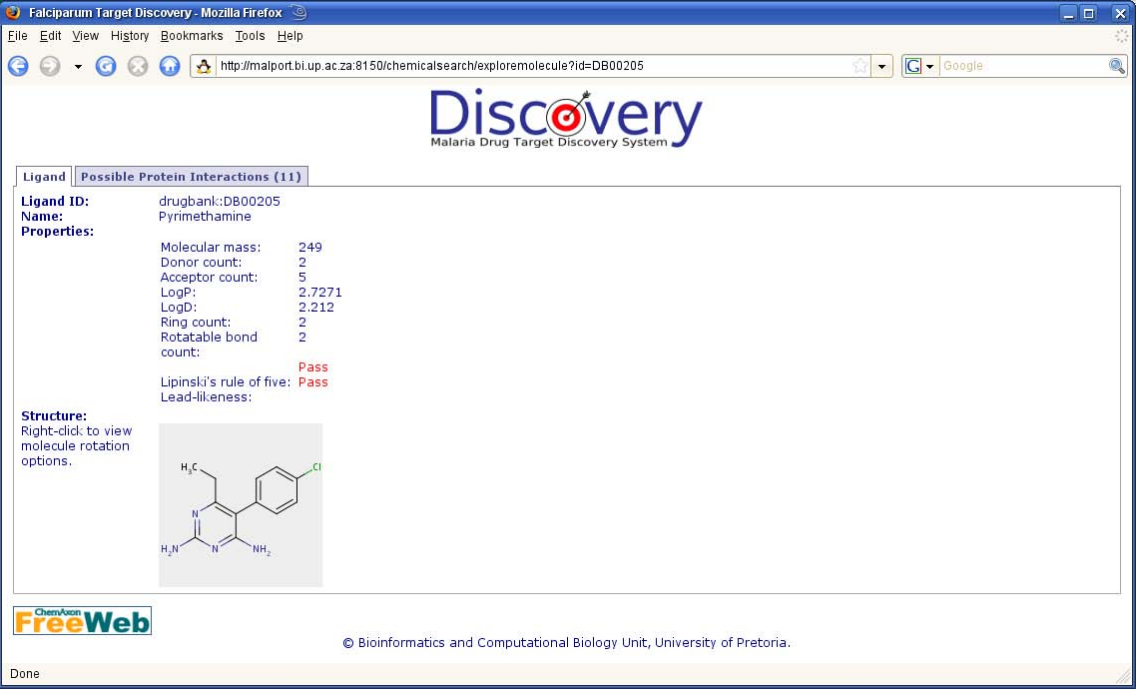
**The Discovery results page for a chemical search, showing the compound properties and protein interaction tab**.

**Figure 5:**
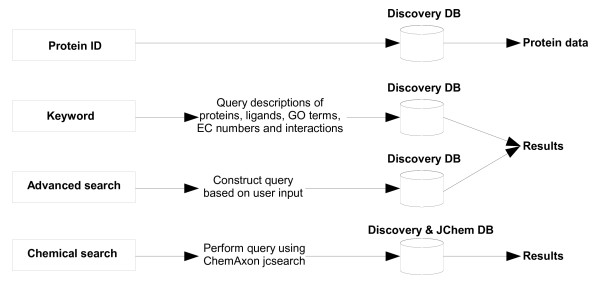
**A representation of the different types of searches available in the Discovery system**.

When approaching the search from the ligand perspective, the user may search by ligand keyword, or by drawing a chemical structure using the Marvin applet. Different methods are then available to search with the structure against ligands from KEGG, PDB and DrugBank using the ChemAxon JChem Base functionality. Results are displayed as a JChem table, and the user may select a compound to view further information. A view is then provided of the compound's structure, ADMET properties as pre-calculated in JChem Base and possible interacting proteins based on KEGG annotations, and BLAST results against PDB and DrugBank (Figure 4). The query processes followed for the different types of searches described above are summarized in Figure 5.

### A sample investigation: *P. falciparum *DHFR-TS

Dihydrofolate reductase-thymidylate synthase (DHFR-TS) was selected to demonstrate the data displayed in the system, as it is well annotated and an established drug target. DHFR-TS occurs as a bifunctional protein in malaria, with both the DHFR and TS proteins fused together as a single polypeptide. Dihydrofolate reductase (DHFR with PlasmoDB id PFD0830w) is inhibited by the class II antifolates, including drugs such as pyrimethamine and proguanil, a prodrug of cycloguanil. All the molecules in the antifolate drug family currently in use were discovered during the Second World War in the 1940's, and since then no new anti-malarial antifolates have been developed that reached phase I/II clinical trials [31]. One of the major causes of antifolate resistance is point mutations in DHFR with a resulting decrease in binding affinity of the inhibitor to the enzyme. Known inhibitors of *P. falciparum *DHFR include antifolate such as pyrimethamine, proguanil, aminopterin [32], methotraxate [33], 2-amino-1,4-dihydro-4,4,7,8-tetramethyl-s-triazino(1,2-a) benzimidazole and Trp-P-2 [34]. Aminopterin and methotrexate inhibit virtually all DHFR enzymes, including *P. falciparum *and the human DHFR, and although these drugs cannot be used to treat malaria because of the possible toxicity of these particular drugs to the human host, precursors thereof may be used to treat malaria [21]. A third generation antifolate compound, dihydrotriazene (WR99210 or BRL 6231) was also recently found to inhibit *P. falciparum *DHFR. WR99210 is different from the resistance-compromized pyrimethamine in that it has a flexible side-chain that binds tightly with not only the wild-type enzyme, but also the mutant *P. falciparum *DHFR, while still retaining anti-malarial activity [35].

*Plasmodium falciparum *DHFR-TS was selected in the Discovery system by searching with the keyword dihydrofolate, and selecting the entry 'PFD0830w bifunctional dihydrofolate reductase-thymidylate synthase'. On the summary page the EC annotation shows the two enzyme domains in the protein, EC:1.5.1.3, corresponding to the dihydrofolate reductase enzymes and EC:2.1.1.45 corresponding to the thymidylate synthases, and links to KEGG are provided. The GO annotations include GO:0006231 (dTMP biosynthetic process), GO:0006545(glycine biosynthetic process), GO:0006730(one-carbon compound metabolic process), GO:0009165 (nucleotide biosynthetic process), GO:0004146 (dihydrofolate reductase activity) and GO:0004799 (thymidylate synthase activity). Orthologs, based on OrthoMCL analysis, are indicated as: *A. gambiae *AGAP010457-PA with no description, three human enzymes, namely ENSP00000314727, ENSP00000314902 and ENSP00000315644, all three of which are thymidylate synthases, a *P. berghei *protein PB000415.02.0 and a *P. chabaudi *protein PC000873.02.0, both putative bifunctional dihydrofolate reductase thymidylate synthases. The *P. yoelii *protein PY04370, a putative thymidylate synthase, was also returned. It should be noted here that the ortholog prediction encounters a special case with bifunctional proteins, as a protein may only occur in one OrthoMCL cluster, which in effect groups bifunctional proteins according to only one of the domains. In this case, the human and mosquito orthologs were for the TS domain of the bifunctional enzyme. The T-Coffee alignment of these proteins is shown in the JalView applet, enabling further manipulations of the sequences.

When considering the summary of the InterPro results, the first featured annotation common to all species is a BLAST hit (TS) against ProDom, a database of protein domain families automatically generated from the UniProt Knowledge Database. Gene3D G3DSA:3.40.430.10 is present in all the Plasmodium species, but not in human or mosquito. HMMPfam returned two hits as to be expected for the bifunctional sequence, namely PF00186, described as DHFR 1, and PF00303 described as Thymidylat synt.

In human and mosquito, only the TS domain is associated with this ortholog group, as DHFR and TS occur separately in these species. HMMPIR hit PIRSF000389 identifies the protein as a bifunctional DHFR-TS in all the Plasmodium species. PatternScan returns two hits, PS00075 and PS00091, identifying DHFR 1 and thymidylate synthase in the Plasmodium species, except for *P. chabaudi *that does not show the DHFR domain (probably due to the shorter sequence). Only the TS domain is shown in human and mosquito. ProfileScan returns ID PS51330, identifying the Plasmodium proteins as DHFR 2. Superfamily identifies both parts of the bifunctional protein in the Plasmodium species as SSF55831 (TS) and SSF53597 (DHFR). HMMTigr TIGR03284 (TS) and Gene3D G3DSA:3.30.572.10 (TS) are present in all species included. This comparison highlights possible areas that may be investigated to identify parasite-specific features in the protein.

Three ModBase models are reported for this protein, based on DHFR-TS from *Leishmania major *(PDB template 1qzf), *P. falciparum *(PDB template 1j3q) and TS from *Pneumocystis carinii *(PDB template 1f28). A range of high-scoring PDB BLAST hits over both DHFR and TS are returned. PFD0830w is predicted through KEGG annotations and BLAST searches to potentially bind with more than a hundred ligands, including the well known inhibitors of DHFR. When searches were performed using common chemical compound names or structures such as pyrimethamine or proguanil, the bifunctional DHFR-TS from *P. falciparum *was listed as a possible interacting protein. No protein-protein or host-pathogen interactions were predicted for this protein.

This sample investigation effectively highlights the main features of DHFR-TS as described in the Discovery system, including possible orthologs, protein motifs and domains anda set of ligands possibly interacting with this protein.

## Conclusion

The Discovery system currently provides basic and advanced interfaces to search protein properties in the resource, as well as a chemical search interface for ligands. Results include orthology, protein features, metabolic information, structural information, and interaction information. Of specific interest is the availability of relationships between proteins and potential interacting ligands, together with ADMET-related properties for the ligands. The system currently has some shortcomings such as the lack of statistical measures for predicted protein-ligand and protein-protein interactions. This could be improved by incorporating sequence conservation across similar sequences for scoring purposes. Also, the prediction of host-pathogen protein-protein interaction does not yet take similarity, sub-cellular localization and properties such as PEXEL motifs and transmembrane regions into account. Additionally, in the special case of bifunctional proteins, these are currently clustered only with the ortholog of the best-matching domain by OrthoMCL, and alternative approaches need to be investigated for bifunctional matching. These and other shortcomings will be addressed in future releases, yielding futher improvements to the system.

It is anticipated that the Discovery system will offer an effective starting point where users interested in drug discovery may obtain relevant information about a pre-selected malaria protein, and discover other proteins of interest by searching according to a selection of protein and ligand properties. The system's further development will serve to make it a valuable tool in the malaria drug discovery process.

## Availability



## Competing interests

The authors declare that they have no competing interests.

## Authors' contributions

The orthology view functionality was developed by FJ and TAPDB, the function, metabolism and structure views by FJ, RJK and TAPDB, the protein-protein interactions by CMH, the protein-ligand interaction by TAPDB, CMH, FJ, the host-pathogen interaction view by CJO and the chemical searches by FJ and TAPDB. All authors contributed to the writing of the manuscript.
